# Sexual Debut Ages in Heterosexual Norwegians Across Six Birth Cohorts

**DOI:** 10.1007/s12119-022-10045-z

**Published:** 2022-12-08

**Authors:** Nantje Fischer, Bente Træen, Sven Ove Samuelsen

**Affiliations:** 1 Department of Psychology, University of Oslo, Oslo, Norway; 2 Department of Mathematics, University of Oslo, Oslo, Norway

**Keywords:** Vaginal sex debut age, Oral sex debut age, Anal sex debut age, General population

## Abstract

This study aims to estimate six different sexual debut ages in heterosexual Norwegians in six birth cohorts of the general population in Norway. The results are based on a 2020 national web panel survey of 18–89-year-olds in Norway (n = 4160). There was a general decline in the median debut age from those aged 70+ to those 18–29 (born 1991–2002). Oral sex with a female partner has become increasingly common among men at an earlier age across generations. The same pattern was found in women as well with regard to oral sex with a male partner and receptive anal sex. There was a slight increase in median debut age in 18–29-year-olds for receptive vaginal sex (born 1991–2002). The median debut age for vaginal sex was fairly stable for generations of men and women born after 1950.

## Introduction

Sexuality is shaped by the historical period; sexual debuts, therefore, must be considered by the era individuals were born in and in which they grew up. Important macro-sociological changes have taken place in the post-World War II Norway, which may have influenced sexual debut ages. The post-World War II Norwegians had their sexual intercourse debut at the age of 17–20 years (Stigum et al., 2010), ushering in periods that offered different opportunities to express sexuality, including the timing of the sexual debut age. In the 1950s, new technological interventions revolutionized work in the domestic sphere, and a “youth culture” emerged. Contraception methods were more insecure for unwanted pregnancy (condoms, interrupted intercourse, secure periods, pessary). In Norway, this changed from 1967, when contraceptive pills became available in the market. Undoubtedly, this eased women’s fears of becoming pregnant. In birth cohorts from 1950 onwards, women in Norway and other Nordic countries experienced their first sexual intercourse at an age earlier than that for men (Kontula & Haavio-Mannila, 1995; Lewin et al., 2000; Stigum et al., 2010; Stryhn & Graugaard, 2014). For example, a recent large Danish study of 62,675 adults aged 18–89 years showed that among cohorts born after the 1950s, women’s estimated median age of debut (defined as mutual masturbation, vaginal, oral, or anal sex intercourse) with an individual of the opposite sex has been approximately half a year lower than men’s (Frisch et al., 2019).

During the 1960s and the 1970s, Norway was characterized by social mobility (Ramsøy, 1977) and happy-go-lucky sexual emancipation. Middle-class norms, attitudes, and behavior began to be reflected in larger segments of the population (Træen & Stigum, 1998). Additionally, Norway discovered oil in the North Sea, which led to economic growth. During the 1980s, Norway was characterized by a philosophy of individualism, cultural urbanization, and the fragmentation of public life and life in general (Hylland Eriksen, 1993). Norwegians born in the 1980s could be regarded as products of the social and cultural process of individualization (Hylland Eriksen, 1993), which is best characterized by terms, such as flux, movement, negotiation, and risk (Beck, 1992; Beck et al., 1994; Crook et al., 1992; Featherstone, 1990; Giddens, 1992). Young people are subjected to various types of uncertainties and risks. Sexual emancipation of the 80 s and 90 s came with the fear of human immunodeficiency virus (HIV), acquired immunodeficiency syndrome (AIDS) and a loss of innocence. Sex became dangerous.

In many heterosexuals, sexual intercourse is synonymous with vaginal penetration. This may be why “sexual intercourse” has been seldom defined for survey participants. A recent Norwegian study designed questions for individuals of all sexual orientations (Træen et al., 2016). The questions considered specific sexual actions: that penetration could occur with fingers and objects in addition to the penis, and that in penetration the individual can have an active (insertive) or passive (receptive) role. The median sexual debut ages among heterosexual men aged 18–29 years (born 1984–1995) was 18.3 years for insertive vaginal sex, 18.7 years for oral sex with a female partner, and 27.1 years for insertive anal sex. Considering that women usually have their first intercourse with an older male partner (2–3 years), the median debut ages for heterosexual women was 16.8 years for receptive vaginal sex, 17.5 years for oral sex with a male partner, and receptive anal sex at 22.7 years. In comparison, in 2009, the age of first heterosexual intercourse, assumed vaginal penetration, was estimated at 17.0 years and 17.9 years among girls and boys, respectively (Træen et al., 2011).

Adolescent sexuality is expressed differently today than it was a few decades ago (Plummer, 2019). Many researchers believe that sexualization of the youth has increased in recent years, as shown in popular culture and social media (Ringrose, 2016). Internet pornography is easily accessible, queer sexuality is subject of numerous websites, social media is extensively used, sexual and gender diversity is being encouraged (Pedersen et al., 2021). New methods to secure hormonal anticonception are available (Træen & Fischer, 2021), and evidence suggests greater sexual diversity and experimentation (Kvalem et al., 2014; Træen et al., 2021). In Norway, a large representative study among adolescents aged 13–19 years showed a decline in age for median sexual debut between 1992 and 2002 from 18.5 to 18.0 years for boys and from 17.7 years to 16.7 years for girls (Pedersen & Samuelsen, 2003). In contrast, recently Pedersen et al. (2021) found that the sexual debut age in adolescents rose in the Norwegian capital, Oslo. This trend coincides with a pattern of less problematic behavior, lower alcohol consumption, and an increase in the proportion of the population with immigrant backgrounds (Rogne et al., 2019). Immigrant backgrounds seemed to lower the likelihood of sexual intercourse, whereas consuming alcohol and being active on social media increased the likelihood of coital debut (Pedersen et al., 2021). However, as adolescents from the capital seem to differ in several ways from those in other areas of Norway (Bakken, 2019), the trend of an increasing sexual debut might not be representative of Norwegian adolescents in general. Recent data on people’s sexual debuts in different birth cohorts is important as it may help to guide public health policy development and dissuade myths about how early people experience different sexual debuts (vaginal, oral and anal sex). To the best of our knowledge, there are no recent studies estimating men’s and women’s experiences with vaginal debut or trends in other types of first sexual experiences in Norway. As humans are inherently social with a need to have their sexual experiences confirmed as “common” or “normal” (Træen & Lewin, 2008), the current study aims to provide the latest estimates of the median ages of vaginal, oral, and anal debuts, across six age cohorts. Finally, to form a preventive perspective, changing trends in sexual debut should be carefully monitored as young age at first intercourse has been shown to be associated with many risk-taking behaviors (Olesen et al., 2012; Stryhn & Graugaard, 2014).

## Methods

### Participants

In March 2020, 11,685 Gallup Panel (a subsidiary of Kantar in Norway) members were randomly invited to participate in an online survey on sexuality. Of those asked to participate, 4,160 individuals aged 18–89 years completed the questionnaire, resulted in a response rate of 35.6% (7525 individuals did not complete the survey). Approximately every second participant (51%) completed the online survey using their mobile phones.

### Recruitment

For this study, the Norwegian Gallup collected data based on its web panel, which has approximately forty thousand active members (https://www.galluppanelet.no/). Norwegian members of the Kantar Gallup Panel were randomly recruited through national phone registries; thus, there is no possibility of self-recruitment. The Gallup Panel represents Norway’s population of Internet users, which, in turn, reflects 98% of the population with access to Internet (see http://www.medienorge.uib.no/english/). Gallup Panel members were contacted regularly to complete online questionnaires. To motivate participation, Kantar developed a carefully planned incentive programme. Although small incentives were provided (e.g., lotteries, occasional surprises of varied quality), these were insufficient to motivate study participation.

### Ethics

Participation in the study was voluntary. Participants were guaranteed anonymity, they were able to skip questions and to withdraw from the study at any time without giving any reason. The research complied with the Personal Data Act and the guidelines of the Norwegian Data Protection Authority. It followed the ethical guidelines developed for market and poll organization surveys (Norway’s Market Research Association and the European Society for Opinion and Market Research [ESOMAR]).

### Sample Characteristics

Approximately half of the respondents were male (52.4%, *n* = 2181) and 47.6% female (*n* = 1979). Female participants were somewhat younger than their male counterparts (mean__women_ = 44.4 years (*SD* = 16.8) and mean__men_ = 48.4 years (*SD* = 17.1)).

At least 59.5% participants had no religious affiliation, 18.1% reported being Christian with no denomination, 0.7% Roman Catholic, 17.1% Protestant, 2.8% Baptist/Methodist/Evangeline, 0.7% Muslim, and 1.2% “other”. As many as 63.4% reported being married/cohabiting/in a registered partnership, 25.4% unmarried, 8.4% separated or divorced, and 2.8% widowed. Approximately 60% percent of respondents claimed that they were not religious. Most respondents lived in urban areas (56.8%), 26.8% in smaller towns, and 16.3*%* in rural areas. Participants had the following education levels: Schooling, 0.8% (6–8 years), 4.6% (9–10 years), 30.4% (12–13 years). About 41.4% had bachelor’s degree or similar educational qualifications and 22.8% had master’s degree, Ph.D., or similar qualifications. Of all the participants, 5.7% stated that they had never engaged in sexual activity with their partners.

Most participants identified their sexual identity as heterosexual (1984 males, 1831 females), 2.6% as homosexual/lesbian (83 males, 24 females), 3.3*%* as bisexual (60 males, 73 females), 0.6*%* as asexual (11 males, 13 females), and 0.1% as “other” (2 males). Therefore, 156 males and 110 females could be classified as queer and 1984 males and 1831 females as straight. However, across the six birth cohorts, the number of respondents that did not identify as heterosexual was low. Therefore, it was decided to conduct debut age analyses only among heterosexuals.

### The Questionnaire

The questionnaire comprised socio-demographic questions. In addition, questions adapted from the British NATSAL-3 study (Mercer et al., 2013; Mitchell et al., 2013), and German GeSid survey (https://gesid.eu/studie/) were included. Questions on sexual debut age were used in a previous Norwegian study (Træen et al., 2016). The average time to complete the survey was 15 min.

### Measures

**Debut ages** were measured by the questions “How old were you the first time you experienced any of the following (if you do not have the experience please write 99): ‘Vaginal sex where another person penetrated you with fingers, penis, or an object,’ ‘Vaginal sex where you penetrated another with fingers, penis, or an object,’ ‘Oral sex with a woman (you licked or sucked her genitals),’ ‘Oral sex with a man (you licked or sucked his genitals),’ ‘Anal sex where another person penetrated you with fingers, penis, or an object,’ and ‘Anal sex where you penetrated another with fingers, penis, or an object’.” Participants who responded “no” to the question “Have you ever had sex with a partner” were coded 99.

**The birth cohort** was assessed by the year of birth and subsequently recoded into six age group categories: 1931–1950 (≥ 70 years) (1), 1951–1960 (60–69 years) (2), 1961–1970 (50–59 years) (3), 1971–1980 (40–49 years) (4), 1981–1998 (30–39 years) (5), and 1999–2002 (18–29 years) (6).

### Statistical Analysis

We used SPSS/PC version 26 to conduct statistical analyses. Survival analysis was conducted to compare sexual debuts between men and women in the six birth cohorts. At the time of data collection, not all respondents had experienced the various sexual activities under study. Therefore, data was treated as right-censored survival or, (more generally) time to event data. Sexual debut is an event in one’s life history and studies the timing of this event, therefore it relies on life history analysis (Stigum et al., 2010). The term used to study sexual debut as a time-to-event in a person’s life history is “survival analysis.” The data’s main feature regarding time of sexual debut was that the event was not observed for all participants. When event times were either exactly observed or exceeded the observational time, we named it “right censored data.” Since some participants in our study did not have sexual debut, the data was treated as “right-censored survival” (or more generally, as time-to-event) data.

The probability of (not) having experienced each sexual action before different ages could be estimated using Kaplan–Meier curves (Hosmer & Lemeshow, 1999). The median debut age was at the point the curve crossed 0.5. Differences between the different birth cohorts were tested using log-rank tests. Debut ages were reported throughout the year in the questionnaire. However, it was unlikely that the respondents had sexual debuts on their birthdays. Software for Kaplan–Meier estimators typically treats the data as if all events occurred on the respondents’ birthdays. It is more likely that the events occurred somewhere in the age intervals of one year. Random numbers between zero and one were added to the censored event times to adjust for such times in the data (Hosmer & Lemeshow, 1999; Kleinbaum, 1997; Samuelsen et al., 2005).

## Results

Table 1 shows the percentages of heterosexual male and female participants across the six birth cohorts reporting lifetime experiences of different sexual debuts.

**Table 1:** Lifetime experience of sexual debuts among Norwegian heterosexuals (crosstabulation analysis; percent and Chi-square-test)

	Birth cohort (age group)						
	1931–1950 (70+ yrs)	1951–1960 (60–69 yrs)	1961–1970 (50–59 yrs)	1971–1980 (40–49 yrs)	1981–1990 (30–39 yrs)	1991–2002 (18–29 yrs)	Sign
*Women*							
Receptive vaginal sex (fingers, penis or an object)	70.6	76.0	80.3	89.2	87.2	83.9	χ^2^ = 40.7, 5 d.f., *p* < 0.001
Insertive vaginal sex (fingers, penis or an object)	6.1	6.7	10.3	13.8	14.7	15.4	χ^2^ = 21.9, 5 d.f., *p* < 0.001
Oral sex with a female partner	3.3	4.1	6.3	8.6	9.3	6.6	χ^2^ = 11.8, 5 d.f., *p* = 0.037
Oral sex with a male partner	46.7	61.8	73.0	84.3	81.6	80.3	χ^2^ = 125.7, 5 d.f., *p* < 0.001
Receptive anal sex (fingers, penis or an object)	17.2	27.7	43.3	61.9	55.5	49.0	χ^2^ = 139.1, 5 d.f., *p* < 0.001
Insertive anal sex (fingers, penis or an object)	2.2	5.2	13.0	21.0	19.2	12.9	χ^2^ = 59.5, 5 d.f., *p* < 0.001
N =	180	267	300	268	375	441	
*Men*							
Receptive vaginal sex (fingers, penis or an object)	–	–	–	–	–	–	
Insertive vaginal sex (fingers, penis or an object)	64.5	79.5	79.6	84.8	84.7	73.2	χ^2^ = 57.5, 5 d.f., *p* < 0.001
Oral sex with a female partner	55.7	75.8	80.4	82.8	81.3	70.7	χ^2^ = 91.5, 5 d.f., *p* < 0.001
Oral sex with a male partner	1.5	2.2	5.5	5.6	5.2	4.8	χ^2^ = 12.7, 5 d.f., *p* < 0.05
Receptive anal sex (fingers, penis or an object)	1.2	4.8	12.5	17.9	13.5	11.3	χ^2^ = 63.1, 5 d.f., *p* < 0.001
Insertive anal sex (fingers, penis or an object)	12.5	22.8	42.0	54.3	49.9	44.3	χ^2^ = 181.1, 5 d.f., *p* < 0.001
N =	327	273	383	302	385	314	

Receptive vaginal sex can only be experienced by women; and this experience was reported by a majority, and there was a statistically significant difference across birth cohorts (*p* < 0.001). A large majority of men reported experiencing insertive vaginal sex; the number was more for those born after 1950 (< 70 years). A significantly higher percentage of women in the younger birth cohort reported that they had inserted a finger, penis, or object into another person’s vagina (both *p* < 0.001). Less than one in 10 heterosexual women reported having an insertive vaginal intercourse with another woman. Notably, there was a steady increase across birth cohorts of women who had experienced insertive vaginal sex. This experience was most common among heterosexual women aged 18–29 years, 15.4% indicated that they had engaged in insertive vaginal sex with another woman (*p* < 0.001).

Oral sex with a female partner was less common among heterosexual men ≥ 70 years than among younger heterosexual men (*p* < 0.001). Except for the birth cohort of 1931–1960 (60+ years), approximately 5% of heterosexual men reported that they had oral sex with a male partner (*p* < 0.01). A significantly higher percentage of younger (84.3% aged 40–49 years) than older (46.7% aged 70+ years) women participants reported experience of oral sex with a man (*p* < 0.001).

The proportion of women who had a lifetime experienced of receptive anal sex (fingers, penis or an object) was the highest among those born between 1971 and 1980 (40–49 years, 61.9%). It was the lowest among those born between 1931 and 1950 (70+ years, 17.2%). The proportion of men who experienced insertive anal sex followed this pattern as well.

The median debut ages were estimated for different sexual experiences across the six birth cohorts (Table 2). To obtain medians, the estimated proportion of an event must be at least 50%. The number of missing responses varied across the analyses and the time to the event was censored. Debut ages are presented in terms of median and confidence intervals (CI).

**Table 2:** Median debut ages of selected sexual actions among heterosexuals in Norway (Kaplan–Meier survival analysis; median values and confidence intervals in parenthesis)

	Birth cohort (age group)						
	1931–1950 (70+ yrs)	1951–1960 (60–69 yrs)	1961–1970 (50–59 yrs)	1971–1980 (40–49 yrs)	1981–1990 (30–39 yrs)	1991–2002 (18–29 yrs)	Sign
*Men*							
Receptive vaginal sex (fingers, penis or an object)	–	–	–	–	–	–	
Insertive vaginal sex (fingers, penis or an object)	21.2 (20.0–22.5)	18.6 (18.2–19.1)	18.7 (18.4–19.0)	18.9 (18.3–19.5)	18.5 (18.2–18.9)	18.6 (18.2–18.9)	χ^2^ = 54.6, 5 d.f., *p* < 0.001
Oral sex with a female partner	35.6 (24.6–46.7)	21.7 (20.3–23.1)	20.2 (19.7–20.8)	19.4 (18.4–20.2)	19.1 (18.7–19.5)	19.2 (18.3–20.0)	χ^2^ = 158.1, 5 d.f., *p* < 0.001
Oral sex with a male partner	–	–	–	–	–	–	
Receptive anal sex (fingers, penis or an object)	–	–	–	–	–	–	
Insertive anal sex (fingers, penis or an object)	–	–	–	35.2 (26.8–43.6)	37.0 (32.9–41.1)	26.9 (24.9–29.0)	χ^2^ = 308.4, 5 d.f., *p* < 0.001
	(n = 327)	(n = 273)	(n = 383)	(n = 302)	(n = 385)	(n = 314)	
*Women*							
Receptive vaginal sex (fingers, penis or an object)	20.2 (19.3–21.1)	18.1 (17.7–18.4)	17.7 (17.2–18.2)	17.3 (16.9–17.7)	17.4 (17.0–17.8)	17.7 (17.3–18.2)	χ^2^ = 71.6, 5 d.f., *p* < 0.001
Insertive vaginal sex (fingers, penis or an object)	–	–	–	–	–	–	
Oral sex with a female partner	–	–	–	–	–	–	
Oral sex with a male partner	–	25.9 (22.0–29.7)	20.8 (19.2–22.4)	19.4 (18.7–20.0)	18.8 (18.4–19.2)	18.3 (17.9–18.6)	χ^2^ = 283.2, 5 d.f., *p* < 0.001
Receptive anal sex (fingers, penis or an object)	–	–	–	30.2 (26.7–33.7)	30.2 (25.9–34.6)	25.9 (24.6–27.2)	χ^2^ = 293.4, 5 d.f., *p* < 0.001
Insertive anal sex (fingers, penis or an object)	–	–	–	–	–	–	
	(n = 180)	(n = 267)	(n = 300)	(n = 268)	(n = 375)	(n = 441)	

“–” = not available. Tested for statistically significant group differences by means of log-rank test

Regarding insertive vaginal intercourse, men born between 1931 and 1950 had a median debut age of 21.2 years (CI 20.0–22.5), which declined to 18.6 years among men born between 1991 and 2002 (CI 18.2–18.9). Likewise, oral sex with a female partner declined from 35.6 years (CI 24.6–46.7) among men born between 1931 and 1950 to 19.2 years (CI 18.3–20.0) among men born between 1991 and 2002. The median debut age for insertive anal sex could not be calculated for men born prior to 1971 (50 + years). The median debut age for insertive anal sex in men born between 1991 and 2002 (18–29 years) was lowest at 26.9 years (CI 24.9–29.0). Accordingly, men in all birth cohorts accumulated sexual experience in the following order: insertive vaginal sex, oral sex with a female partner, and insertive anal sex.

In women, there were statistically significant differences in the reporting of debut ages across the six birth cohorts on receptive vaginal sex, oral sex with a male partner, and receptive anal sex among women born after 1971 (all *p* < 0.001). There was a decline in receptive vaginal sex debut age for women born between 1931 and 1950 (20.2 years, CI 19.3–21.1), to 17.3 years (born between 1971 and 1980). However, for women born between 1991 and 2002, the median debut age increased to 17.7 years (CI 17.3–18.2). There was a decline in the debut age for oral sex with a male partner as well. The median age of oral sex debut was 25.9 years (CI 22.0–29.7) among women born between 1951 and 1960, and 18.3 years (CI 17.9–18.6) for their counterparts born between 1991 and 2002. Furthermore, there was a decrease in the median debut age for receptive anal sex across the six birth cohorts. The median anal sex debut was 25.9 years (CI 24.6–27.2) among women born between 1991 and 2002. Women in all birth cohorts accumulated sexual experience in the following order: receptive vaginal sex, oral sex with a male partner, and receptive anal sex.

## Discussion

In this study we aimed to estimate, across six age cohorts, the median age when heterosexual men and women have their first vaginal, oral, and anal sex. In both women and men, there was a general decline in the median age of vaginal debut. This finding coincides with an overall decline in age at first intercourse in many western countries (Bajos et al., 2010; Hawes et al., 2010; Lewis et al., 2017; Liu et al., 2015; Stigum et al., 2010). Further, as with other Scandinavian population-based studies, our findings indicate a more or less stabilized sexual debut age among those generations born after 1950 (Danielsson et al., 2012; Hansen et al., 2020; Stryhn & Graugaard, 2014). For example, the median debut age for insertive vaginal intercourse was 18.6 years for men born between both 1951 and 1960 and 1991 and 2002.

Interestingly, among women in the two youngest birth cohorts (those born between 1981 and 2002), we found a slight increase in the median debut age for receptive vaginal sex (from 17.4 years to 17.7 years). This finding points in the same direction as another recent Norwegian study showing an increase in the sexual debut among adolescents in the Norwegian capital between 1996, 2006, 2012 and 2018 (Pedersen et al., 2021). In contrast, a large nationally representative cross‐sectional survey of more than 100 000 women from Norway, Denmark and Sweden indicated a changing trend towards an earlier sexual intercourse debut (median debut age of 16) among women born between 1983 and 1994 (Hansen et al., 2020). Further studies are therefore needed to assess whether recent alterations in age at first intercourse indicate minor fluctuations or actual trend shifts. Different procedures and measures (e.g., operationalization of sexual debut, variations in the classification of birth cohorts, and different sampling and statistical analyses) may explain inconsistencies across studies.

As previously reported, we found gender differences in debut ages (Pedersen & Samuelsen, 2003; Sundet et al., 1992; Teitler, 2002; Træen & Samuelsen, 2007; Træen et al., 2016), with women reporting earlier sexual intercourse debuts than men across all six birth cohorts. Since the beginning of the 1970s, women in Nordic countries had their first sexual intercourse at an earlier age as compared to men (Bozon & Kontula, 1998; Frisch et al., 2019; Kontula & Haavio-Mannila, 1995; Lewin et al., 2000). This was because the social acceptance of adolescent and female sexuality was significantly greater in Nordic countries than in most Western cultures (Lewin, 1991; Træen, 1993). The roots of these differences go back to pre-Christian family formation patterns. The ethnically homogeneous, non-feudal, and sparsely populated Nordic countries, at the outskirts of European civilization, accepted Christianity very late (10th to eleventh century) and retained pre-Christian legislation about marriage for many centuries thereafter (Lewin, 1979). In pre-Christian courtship patterns, it was tolerated for young people to be “lying together” before marriage. Furthermore, Nordic countries were non-feudal societies, which may explain why the prevailing social norms in these countries are traditionally characterized by an ideology of equality for every member of society. This implies that *ideally*, every member of society, irrespective of his/her sub-culture, should be treated and judged equally. Accordingly, expressions of sexuality and social codes that organize them are *ideally* equal for sexual actors. Girls on average enter puberty before boys do; therefore, an earlier coital debut age for heterosexual women than men may be reasonably expected. Moreover, in confirmation with social norms, young women often find a sexual partner that is 2–3 years older (Træen & Samuelsen, 2007). This makes it difficult for male peers to enter a sexual relationship with a partner of the same age and may explain why young men experience their first sexual intercourse later than women.

As with the vaginal debut, there was a decline in the debut age for oral sex with the opposite sex across birth cohorts. In particular, oral sex with a female partner declined from 35.6 years among men in the oldest birth cohort (1931–1950) to 19.2 years among men in the youngest birth cohort (1991–2002). For women the median age fell by 7.6 years, from 25.9 years among those born between 1951 and 1960 to 18.3 years among those born between 1991 and 2002. Although the oral debut with the opposite sex occurred later than the first experience of vaginal sex across all six birth cohorts, the gap between these two types of sexual debuts narrowed substantially among the younger cohorts. This is consistent with the literature, suggesting a shift towards a less traditionally structured temporal sequencing of the first experience of heterosexual vaginal and oral sex (Rissel et al., 2014). As with other studies among Norwegian adolescents, oral sex seems to occur almost simultaneously as vaginal sex (Pedersen & Samuelsen, 2003; Træen & Samuelsen, 2007).

Finally, the median debut age for receptive anal sex in women and insertive anal sex in men was lowest among the youngest birth cohort (women: 25.9 years; men 26.9). This finding is in agreement with other studies, which show an overall increase in people reporting having experienced anal sex. This, in turn, reflects greater experimentation among younger generations (Bajos et al., 2010; Mercer et al., 2013; Vanwesenbeeck et al., 2010).

## Strength and limitations

This study was conducted during the COVID-19 lockdown in March–April 2020. Some individuals may have been less receptive and interested in participating in a study on sexual behavior. We are not sure if this nonresponse bias affected our results. According to a previous study on whether respondents and non-respondents in a Norwegian sexual behavior study showed different patterns of sexual behavior, it is likely that nonresponse is not associated with differences in sexual behavior (Stigum, 1997). Other Nordic studies concluded that nonresponse is fairly random with respect to sexual behavior (Haavio-Mannila & Kontula, 2003; Kontula & Haavio-Mannila, 1995; Lewin et al., 2000). We cannot rule out that the non-respondents have different behavioral patterns from the respondents, but we believe that non-participation is random rather than systematic.

The majority of our respondents had high levels of education, higher than that in the general population. The official 2018 statistics of the Central Bureau of Statistics showed that 34.1% of the Norwegian population aged 16 years or older had a high level of education. Accordingly, our sample was slightly but not severely biased. There may have been a memory bias in the data on sexual debut ages, as this event might have occurred many years ago, especially for the oldest birth cohorts. It is reasonable to assume that a social desirability bias may be present in the reporting of age at sexual debut.

## Conclusions

Apart from minor fluctuations, the median debut age for vaginal sex appeared relatively stable for generations of Norwegian women and men born after 1950. The sexual repertoire of Norwegian women and men aged < 50 years was more varied than that of the older generations.
